# Urinary endotrophin as a biomarker for T cell–mediated rejection-associated fibrogenesis in kidney transplant recipients

**DOI:** 10.1093/ckj/sfaf301

**Published:** 2025-09-30

**Authors:** Firas F Alkaff, Daan Kremer, Daniel G K Rasmussen, Nadja Sparding, Federica Genovese, Morten Karsdal, Wendy A Dam, Marius C van den Heuvel, Martin Tepel, Olivier Thaunat, TransplantLines Investigators, Coby Annema, Stephan J L Bakker, Stefan P Berger, Hans Blokzijl, Frank A J A Bodewes, Marieke T de Boer, Kevin Damman, Martin H de Borst, Arjan Diepstra, Gerard Dijkstra, Caecilia S E Doorenbos, Rianne M Douwes, Michele F Eisenga, Michiel E Erasmus, C Tji Gan, Antonio W Gomes Neto, Eelko Hak, Bouke G Hepkema, Jip Jonker, Frank Klont, Tim J Knobbe, Daan Kremer, Henri G D Leuvenink, Willem S Lexmond, Vincent E de Meijer, Hubert G M Niesters, Gertrude J Nieuwenhuijs-Moeke, L Joost van Pelt, Robert A Pol, Anna M Posthumus, Adelita V Ranchor, Jan Stephan F Sanders, Marion J Siebelink, Riemer J H J A Slart, J Casper Swarte, Daan J Touw, Marius C van den Heuvel, Coretta van Leer-Buter, Marco van Londen, Charlotte A te Velde Keyzer, Erik A M Verschuuren, Michel J Vos, Rinse K Weersma, Stefan P Berger, Jacob van den Born, Stephan J L Bakker

**Affiliations:** Division of Nephrology, Department of Internal Medicine, University of Groningen, University Medical Center Groningen, Groningen, The Netherlands; Division of Pharmacology and Therapy, Department of Anatomy, Histology, and Pharmacology, Faculty of Medicine Universitas Airlangga, Surabaya, Indonesia; Division of Nephrology, Department of Internal Medicine, University of Groningen, University Medical Center Groningen, Groningen, The Netherlands; Nordic Bioscience, Herlev, Denmark; Nordic Bioscience, Herlev, Denmark; Nordic Bioscience, Herlev, Denmark; Nordic Bioscience, Herlev, Denmark; Division of Nephrology, Department of Internal Medicine, University of Groningen, University Medical Center Groningen, Groningen, The Netherlands; Department of Pathology and Medical Biology, University of Groningen, University Medical Center Groningen, Groningen, The Netherlands; Odense University Hospital, Department of Nephrology, Odense, Denmark; University of Southern Denmark, Institute of Molecular Medicine, Cardiovascular and Renal Research, Odense, Denmark; Hospices Civils de Lyon, Hôpital Edouard Herriot (Edouard Herriot Hospital), Service de Transplantation, Néphrologie et Immunologie Clinique, Lyon, France; University of Groningen, University Medical Center Groningen, Groningen, The Netherlands; Division of Nephrology, Department of Internal Medicine, University of Groningen, University Medical Center Groningen, Groningen, The Netherlands; Division of Nephrology, Department of Internal Medicine, University of Groningen, University Medical Center Groningen, Groningen, The Netherlands; Division of Nephrology, Department of Internal Medicine, University of Groningen, University Medical Center Groningen, Groningen, The Netherlands

**Keywords:** biomarkers, collagen α3(VI), endotrophin, graft rejection, kidney transplantation

## Abstract

**Background:**

Endotrophin, a C-terminal pro-collagen type VIα3 fragment, has been shown to correlate with kidney interstitial fibrosis, kidney outcome measures and survival in various kidney diseases and kidney transplantation. In this study we investigated whether endotrophin is associated with fibrogenesis in T cell–mediated rejection (TCMR), allowing its use as a non-invasive biomarker.

**Method:**

Plasma endotrophin and urinary endotrophin (indexed for creatinine) were measured in samples from a cross-sectional study among kidney transplant recipients (KTRs) who underwent indication biopsy after transplantation and enrolled in TransplantLines Biobank and Cohort Study. Endotrophin was measured using the nordicPRO-C6 enzyme-linked immunosorbent assay. Blood and urine were collected on the day of biopsy. In a subset of patients, the biopsy was stained for endotrophin and T cells.

**Results:**

A total of 149 KTRs were included in the analyses. Of them, 48 (32.2%) had TCMR (either borderline, acute, chronic or mixed). Higher urinary endotrophin levels were associated with increased odds of TCMR and the association remained significant after adjustment for other potential confounders, including plasma endotrophin [adjusted odds ratio per doubling 1.38 (95% confidence interval 1.11–1.72), *P* = .004]. In contrast, higher plasma endotrophin levels were not associated with increased odds of TCMR. T cell density in the biopsy was associated with endotrophin-positive myofibroblasts (ρ = 0.61, *P* = .045), urinary endotrophin (ρ = 0.67, *P* = .017) and interstitial fibrosis and tubular atrophy (ρ = 0.61, *P* = .048).

**Conclusion:**

These data indicate the potential use of urinary endotrophin as a non-invasive biomarker for fibrogenesis in the context of TCMR.

## Graphical Abstract

**Graphical Abstract:**
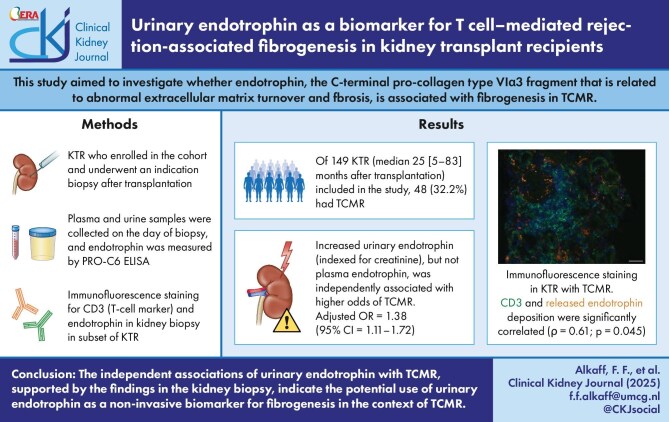

KEY LEARNING POINTS **What was known:**

- Rejection remains one of the main problems in kidney graft survival.
- To date, histological examination of a kidney biopsy is the gold standard for diagnosing graft rejection. However, a kidney biopsy is an expensive and invasive procedure with a risk of complications.
- Endotrophin, a proteolytic fragment from the collagen type VI α3 chain that is cleaved off upon secretion from the producing cell, which is related to abnormal extracellular matrix turnover and fibrosis, has been shown to be linked to interstitial fibrosis and tubular atrophy (IFTA) and also independently associated with short- and long-term graft outcomes.

**This study adds:**

- Urinary endotrophin, but not plasma endotrophin, is independently associated with higher odds of having T cell–mediated rejection in kidney transplant recipients undergoing indication biopsy.
- In the kidney biopsies, T cell density correlates with endotrophin density, urinary endotrophin concentration and IFTA score. Furthermore, both T cell and endotrophin densities correlate with kidney function.

**Potential impact:**

- The independent associations of urinary endotrophin with T cell–mediated rejection, supported by kidney biopsy findings, suggest a potential role of urinary endotrophin as a non-invasive biomarker for fibrogenesis in the context of T cell–mediated rejection.

## INTRODUCTION

Kidney transplantation remains the preferred treatment for end-stage kidney disease since it has the most favourable outcomes and is the most cost-effective kidney replacement therapy modality [1, 2]. In recent decades, the short-term outcomes of kidney transplantation have greatly improved while the long-term outcomes remain a major challenge [3, 4]. One of the leading clinical problems limiting long-term graft survival is rejection [5–7].

In the current clinical setting, graft rejection is suspected in kidney transplant recipients (KTRs) with unexplained increases in serum creatinine and/or proteinuria. However, both lack sensitivity and specificity for detecting rejection [8, 9]. Thus a kidney biopsy is required to make the diagnosis. But a kidney biopsy is impractical, as it is an expensive and invasive procedure with a risk of complications, prone to sampling error and not eligible for the performance of serial measurements [8]. Thus there is a need for sensitive and specific non-invasive biomarkers for rejection.

There are two types of rejection: T cell–mediated rejection (TCMR) and antibody-mediated rejection (ABMR). Although TCMR and ABMR can occur concurrently, the pathogenesis of TCMR is different from that of ABMR. The activated immune pathways, the mediating cytokines and chemokines involved in the disease pathogenesis, as well as the type of cells targeted by the immune response, are different [10]. Histologically, TCMR may present with tubulitis, interstitial inflammation and interstitial fibrosis and tubular atrophy (IFTA). In contrast, ABMR may present with microvascular inflammation, acute thrombotic microangiopathy or C4d deposition in peritubular capillaries [11].

Endotrophin is a proteolytic fragment from the C-terminus of the collagen type VI α3 chain (ColVIα3) that is cleaved off upon secretion from the producing cell [12]. Endotrophin has been shown to be positively associated with IFTA in patients with kidney diseases [13, 14]. In the KTR population, endotrophin has also been shown to be linked to the IFTA score [15] and also independently associated with graft outcomes and patient survival [16–18]. Histological evaluation showed that endotrophin is present in human kidneys with fibrotic foci but barely in healthy kidneys at the same site of collagen type VI deposition [19], and

that intense collagen type VI deposition is observed in fibrous lesions in KTRs with chronic rejection [20].

Given that inflammation and tubulitis in IFTA are included in the diagnosis criteria for TCMR [11], and that endotrophin has previously been shown to be associated with IFTA and graft outcomes, we aimed to investigate whether circulating and urinary endotrophin is associated with fibrogenesis in TMCR and might therefore be useful as a non-invasive biomarker.

## MATERIALS AND METHODS

This study was reported following the Strengthening the Reporting of Observational Studies in Epidemiology (STROBE) guidelines (Supplementary Table 1) [21].

### Study design and population

This cross-sectional study used data from the ongoing prospective TransplantLines Biobank and Cohort Study (NCT03272841). The study was conducted in accordance with the Declaration of Helsinki and the Declaration of Istanbul and the study protocol was approved by the Institutional Review Board of the University Medical Center Groningen (UMCG) (METc 2014/077) [22]. The current study used data and measurements from KTRs who underwent an indication biopsy procedure after transplantation between 2016 and 2023. Within the UMCG, indication biopsies are usually performed when kidney function fails to start within 5–7 days after transplantation, there less than expected kidney function shortly after transplantation or in cases of unexplained deterioration of kidney function and/or increased urinary protein excretion. If a KTR had undergone more than one biopsy, only the first biopsy’s data and measurements were used. Furthermore, since previous data indicate that endotrophin was transiently elevated within the first week after transplantation [18], we only included biopsies that were taken at least 1 week after transplantation.

### Clinical and laboratory parameters

Relevant information, including the biopsy report, was extracted from the electronic medical records. Blood and spot urine samples were collected on the day of the biopsy but before the procedure and immediately stored in −80°C freezers (Panasonic, ’s-Hertogenbosch, The Netherlands). Plasma and urinary endotrophin were measured using the nordicPRO-C6 enzyme-linked immunosorbent assay (ELISA) kit (Nordic Bioscience, Herlev, Denmark). The antibody in the kit detects the C-terminal domain of ColVIα3, which includes the C5 domain (endotrophin) that is released upon deposition in the extracellular matrix. The urinary endotrophin level was indexed for creatinine. Creatinine was determined by using the Jaffe reaction (MEGA AU510, Merck Diagnostica, Darmstadt, Germany) and the estimated glomerular filtration rate (eGFR) was calculated using the creatinine-based Chronic Kidney Disease Epidemiology Collaboration (CKD-EPI) formula. Other clinical chemistry assays were performed using routine spectrophotometric methods (Roche Diagnostics, Basel, Switzerland). As we used spot urine samples, proteinuria was expressed as g/10 mmol creatinine. This index is an estimate of urinary protein excretion in g/day in a 24-h urine collection.

### Biopsy evaluation

Categorization of the IFTA score and diagnosis of rejection was made by the nephropathologists at the UMCG based on the Banff classification that was applicable at the time the biopsy was taken. As we included biopsies that were taken between 2016 and 2023, the Banff classification 2015 [23], 2017 [24] or 2019 [11] was used. KTRs with either acute or chronic TCMR were included in the TCMR group. Since borderline rejection is considered highly suspicious for acute TCMR [11], it was also considered as TCMR. Furthermore, because the pathogenesis of TCMR is different from ABMR [10], KTRs with mixed rejection (both TCMR and ABMR) were also included in the TCMR group. KTRs with other histologic injury phenotypes other than TCMR [pure ABMR, transplant glomerulopathy or BK virus–associated nephropathy (BKVAN)] or KTRs without major histologic abnormalities were included in the non-TCMR group.

In a subset of KTRs, 4-µm frozen kidney biopsy sections were used for immunofluorescent (IF) staining. Sections were double-stained for polyclonal rabbit anti-human antibodies against T cell marker CD3 (A0452, DAKO, Glostrup, Denmark) in combination with the same PRO-C6 monoclonal mouse anti-human antibody (Nordic Bioscience, Herlev, Denmark) employed in the nordicPRO-C6 ELISA and with an antibody detecting the N-terminal cleavage of endotrophin by bone morphogenetic protein-1 (BMP-1) (released endotrophin) (Nordic Bioscience) (Supplementary Table 2). Sequential sections from the same biopsies were stained with polyclonal rabbit-anti-human TEM8 (bs-15583R, Bioss, Woburn, MA, USA) (Supplementary Table 3). IF was visualized using a Leica immunofluorescence microscope (DM4000B equipped with DFX345FX camera and LAS software package, Leica Microsystems, Rijswijk, The Netherlands). A minimum of 4 (median of 8 and maximum of 12) pictures at 100× magnification were taken with identical exposure settings and thereafter quantified with ImageJ software with standardized threshold settings and expressed as the positively stained percentage area. The median value of all pictures per biopsy was used for the analysis.

### Statistical analyses

All data were analysed using R version 4.0.5 (R Foundation for Statistical Computing, Vienna, Austria). For all analyses, a two-sided *P*-value <.05 was considered significant. Visual evaluation of the quantile–quantile plots was used to assess the data distribution. Continuous variables were presented as mean ± standard deviation (SD) for the normally distributed variables and median [interquartile range (IQR)] for the skewed variables. Categorical variables were presented as frequency (percentage). Differences in baseline characteristics between KTRs with and without TCMR were tested by independent *t*-test, Mann–Whitney U-test and chi-squared test as appropriate.

Logistic regression analyses were used to evaluate the association of plasma and urinary endotrophin levels with the odds of TCMR. Collinearity was checked by calculating the variance inflation factor of each model, and none had a variance inflation factor >5. Several adjustments were performed to account for the effect of potential confounders. In model 1, analyses were adjusted for age and sex. In model 2, analyses were further adjusted for time after transplantation. In model 3, analyses were further adjusted for eGFR. In model 4, analyses were further adjusted for proteinuria. In model 5, analyses were further adjusted for the plasma C-reactive protein (CRP) concentration. Additionally, for urinary endotrophin, the analyses from model 5 were further adjusted for plasma endotrophin level. To account for missing data other than plasma or urinary endotrophin, multiple imputations using fully conditional specification were performed.

Potential effect modifications by age, sex, eGFR and proteinuria were tested by fitting both main effects and their cross-product terms in the full model. Additionally, potential effect modifications by plasma endotrophin were also assessed for urinary endotrophin. Sensitivity analyses were performed by excluding KTRs who underwent biopsy within 1 month and 3 months after transplantation. Additionally, since BKVAN is also associated with a tubulointerstitial response to injury, we conducted another sensitivity analysis by excluding KTRs with BKVAN (defined as having a positive SV40 staining in the biopsy in combination with tubulointerstitial inflammation). Along with BKVAN, other histologic injury phenotypes may also cause tubulointerstitial injury. Therefore, we conducted sensitivity analyses by additionally excluding KTRs with other histologic injury phenotypes. Lastly, since native kidneys may also produce endotrophin, we conducted sensitivity analyses by stratifying the KTRs based on the presence of residual kidney function at the time of transplantation (defined as having a residual urine output >200 ml/day).

As a secondary analysis, we explored the risk‐prediction ability of plasma and urinary endotrophin for TCMR. In the current clinical setting, we mainly rely on serum creatinine and proteinuria values. Therefore, we investigate whether adding plasma or urinary endotrophin on top of serum creatinine and proteinuria improves the model risk-prediction ability. For this, we used the Akaike information criterion (AIC) to evaluate which model has the better model fit, where lower AIC values indicate a better model fit. We also performed an *F*‐test to evaluate whether the difference between risk-prediction ability models was statistically significant. Finally, a receiver operating characteristics (ROC) curve was generated for the reference model before and after inclusion of plasma or urinary endotrophin and the area under the curve (AUC) was calculated for both curves.

## RESULTS

### Baseline characteristics

The flow chart of the study population selection is presented in Supplementary Fig. 1. There were 149 KTRs [mean age 53 ± 15 years, 38.3% male, median 25 months (IQR 5–83) after transplantation, mean eGFR 30.9 ± 15.3 ml/min/1.73 m^2^] included in the analyses (Table 1). Of these, 48 (32.2%) had TCMR with or without humoral rejection components [37 had pure TCMR, 7 had TCMR with ABMR (5 with positive DSA and C4d and 2 with negative DSA and positive C4d) and 4 had TCMR with transplant glomerulopathy]. The remainder of the KTRs had either ABMR (6 KTR, all with positive DSA and C4d), transplant glomerulopathy (21 KTRs) or were without rejection (74 KTRs). The IFTA score and CRP level were significantly higher in KTRs with TCMR (*P* = .041 and *P* = .001, respectively). There was no difference in kidney function or urinary creatinine levels (Table 1).

**Table 1:** Baseline characteristics of the KTRs undergoing indication biopsy.

Characteristics	Total (*N* = 149)	Non-TCMR (*n* = 101)	TCMR (*n* = 48)	*P*-value
Plasma endotrophin level (ng/ml), median (IQR)	15.6 (12.4–21.3)	14.9 (11.8–21.3)	16.4 (13.7–21.2)	.4
Urinary endotrophin:creatinine ratio (ng/mg), median (IQR)	13.3 (3.1–51.6)	8.3 (2.5–37.9)	29.1 (7.6–80.4)	.003
Demographics				
Female, *n* (%)	57 (38.3)	40 (39.6)	17 (35.4)	.8
Age (years), mean ± SD	53 ± 15	52 ± 15	54 ± 16	.6
Time from transplantation (months), median (IQR)	25 (5–83)	27 (4–97)	21 (7–48)	.6
Transplant-related characteristics				
Pre-emptive transplantation, *n* (%)	48 (32.4)	29 (29.0)	19 (39.6)	.3
Anuria prior to transplantation, *n* (%)	42 (28.2)	31 (31)	11 (23)	.3
First kidney transplantation, *n* (%)	107 (71.8)	75 (74.3)	32 (66.7)	.4
Total HLA (A-B-DR) mismatch	3 (2–4)	3 (2–4)	3 (2–5)	.2
Cold ischaemia time (minutes), median (IQR)	199 (157–686)	198 (157–673)	203 (157–752)	.7
Living donor, *n* (%)	85 (57.4)	58 (58.0)	27 (56.2)	1.0
Donor age (years), mean ± SD	51 ± 14	50 ± 13	54 ± 15	.1
Delayed graft function, *n* (%)	29 (19.7)	24 (24.2)	5 (10.4)	.079
Immunosuppressive medication, *n* (%)				
Use of calcineurin inhibitors	131 (94.2)	86 (92.5)	45 (97.8)	.4
Use of proliferation inhibitors	111 (79.9)	76 (81.7)	35 (76.1)	.6
Use of mTOR inhibitors	10 (7.2)	5 (5.4)	5 (10.9)	.4
Laboratory evaluation				
CRP (mg/l), median (IQR)	2.80 (1.00–6.25)	2.25 (0.72–4.85)	4.45 (2.28–9.25)	.001
Serum creatinine (µmol/l), median (IQR)	193 (155–274)	179 (53–265)	211 (158–276)	.2
eGFR (ml/min/1.73 m^2^), mean ± SD	30.9 ± 15.3	32.3 ± 16.4	27.7 ± 12.3	.087
Urinary creatinine (mg/dl), median (IQR)	70 (46–103)	72 (47–110)	65 (42–89)	.1
Urinary protein:creatinine ratio (g/10 mmol), median (IQR)	0.01 (0.00–0.02)	0.01 (0.00–0.02)	0.00 (0.00–0.01)	.064
Biopsy evaluation				
IFTA, *n* (%)				
<10%	60 (43.1)	46 (47.4)	14 (33.3)	.041
10–25%	36 (25.9)	19 (19.6)	17 (40.5)	
26–50%	24 (17.3)	16 (16.5)	8 (19.0)	
>50%	19 (13.7)	16 (16.5)	3 (7.1)	
Transplant glomerulopathy, *n* (%)	25 (16.8)	21 (20.8)	4 (8.3)	.095
Antibody-mediated rejection, *n* (%)	13 (8.7)	6 (5.9)	7 (14.6)	.2
SV40-positive, *n* (%)	5 (0.7)	5 (5.0)	0 (0)	.3

Immunosuppressive medication was missing in 10 (6.7%) subjects, IFTA was missing in 10 (6.7%) subjects, CRP was missing in 29 (19.5%) subjects and protein:creatinine ratio was missing in 13 (8.7%) subjects.

mTOR: mechanistic target of rapamycin.

*P* < .05 was considered statistically significant.

**Figure 1:**
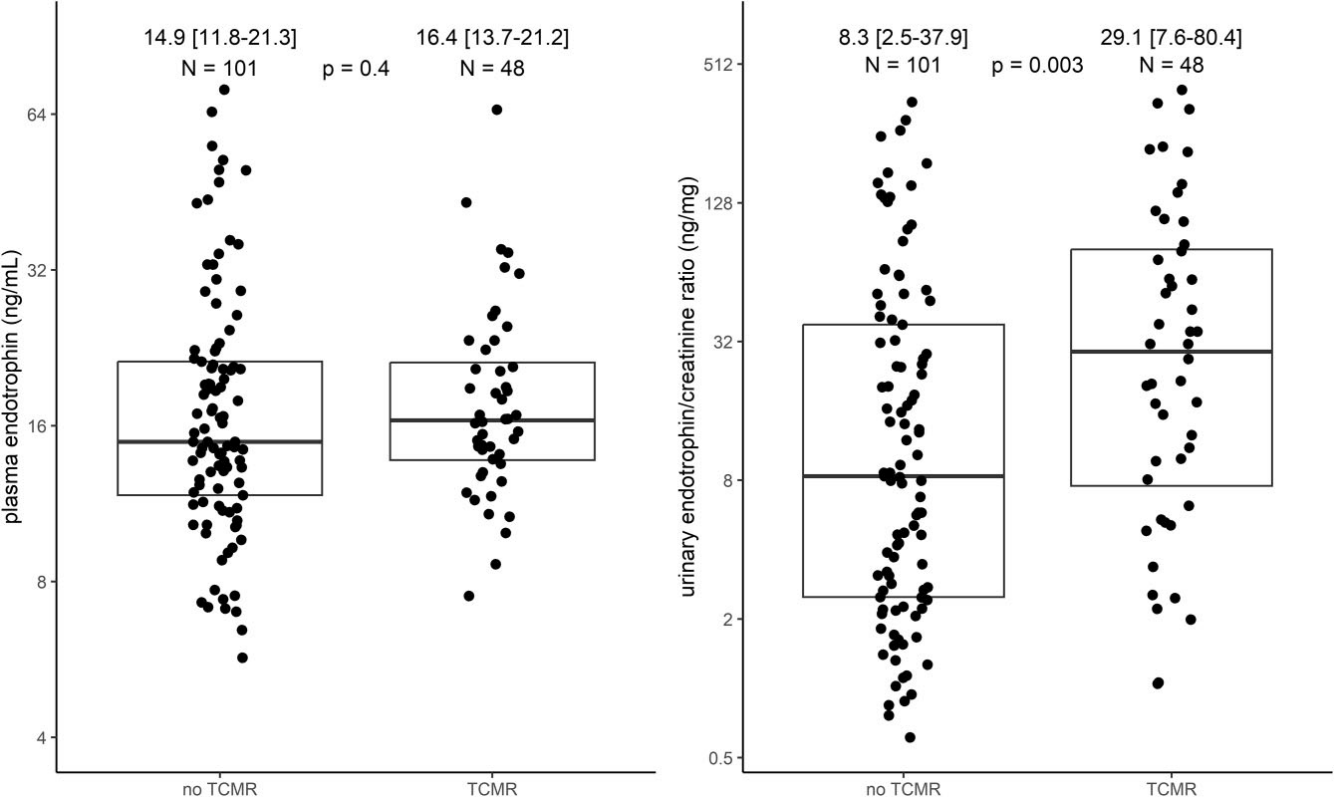
Plasma endotrophin level and urinary endotrophin:creatinine ratio between KTRs undergoing an indication biopsy with and without T cell–mediated rejection. Each dot represents one KTR and the box plot indicates the median (IQR).

Although plasma and urinary endotrophin levels were significantly correlated (Supplementary Fig. 2), plasma endotrophin levels did not differ in KTRs with and without TCMR, whereas urinary endotrophin was significantly higher in KTRs with TCMR (Fig. 1). When grouped further based on the type of rejection, those with pure TCMR had significantly higher urinary endotrophin but similar plasma endotrophin levels than those with no rejection (Supplementary Fig. 3).

### Association of endotrophin with TCMR

Higher plasma endotrophin levels were not associated with increased odds of TCMR. In contrast, higher urinary endotrophin levels were associated with increased odds of TCMR {odds ratio [OR] per doubling 1.25 [95% confidence interval (CI) 1.07–1.45]} in the univariable analysis and the association remained robust independent of adjustment for potential confounders [adjusted OR per doubling 1.28 (95% CI 1.05–1.56)] (Table 2). The association became stronger after further additional adjustment for plasma endotrophin [adjusted OR per doubling 1.38 (95% CI 1.11–1.72), *P* = .004]. There was no indication of effect modification by age, sex, eGFR or proteinuria for the association between plasma or urinary endotrophin and TCMR. There was also no indication of effect modification by plasma endotrophin for the association between urinary endotrophin and TCMR (all *P*_interaction_ > .05).

**Table 2:** Logistic regression analyses of the association of endotrophin with the odds of TCMR.

	Plasma endotrophin		Urinary endotrophin:creatinine ratio	
Model	OR per doubling (95% CI)	*P*-value	OR per doubling (95% CI)	*P*-value
Crude	1.14 (0.71–1.83)	.6	1.25 (1.07–1.45)	.004
Model 1	1.11 (0.69–1.80)	.7	1.24 (1.07–1.45)	.004
Model 2	1.08 (0.66–1.75)	.8	1.22 (1.05–1.42)	.009
Model 3	0.69 (0.34–1.38)	.3	1.24 (1.02–1.50)	.029
Model 4	0.72 (0.35–1.46)	.4	1.28 (1.05–1.56)	.016
Model 5	0.73 (0.35–1.52)	.4	1.28 (1.05–1.56)	.014

There were 48 (32%) KTRs with TCMR. Model 1 was adjusted for age and sex. Model 2 was further adjusted for time after transplantation. Model 3 was further adjusted for the eGFR based on the creatinine-based CKD-EPI formula. Model 4 was further adjusted for log_2_ urinary protein:creatinine ratio. Model 5 was further adjusted for CRP level.

In the first sensitivity analysis, we excluded KTRs who underwent biopsy within 1 month after transplantation (*n* = 13). After excluding these KTRs, the association of urinary endotrophin with the odds of TCMR remained materially unchanged (Supplementary Table 4). In the second sensitivity analysis, we excluded KTRs who underwent biopsy within 3 months after transplantation (*n* = 25). Similarly, the association of urinary endotrophin with the odds of TCMR remained materially unchanged after excluding these KTRs (Supplementary Table 5). Next, we excluded KTRs with BKVAN (*n* = 5) and additionally excluded KTRs who had other histologic injury phenotypes (with or without TCMR) (*n* = 38). The association of urinary endotrophin with the odds of TCMR remained materially unchanged after excluding these KTRs (Supplementary Tables 6 and 7). In the last sensitivity analysis, we stratified the KTRs based on the presence of residual kidney function at the time of transplantation. The level of urinary endotrophin was significantly higher in those without residual kidney function compared with those with residual kidney function [28 ng/mg creatinine (IQR 5–87) versus 10 ng/mg creatinine (IQR 2–38), *P* = .019], suggesting that native kidneys did not make a significant contribution to the urinary endotrophin level. Furthermore, KTRs without residual kidney function had a significantly higher rate of kidney transplantation from deceased donors and delayed and worse graft function (Supplementary Table 8). Urinary endotrophin was associated with the odds of TCMR only in KTRs with residual kidney function and not in KTRs without residual kidney function (Supplementary Table 9).

### Endotrophin and prediction of TCMR

As a secondary analysis, we investigated whether adding plasma or urinary endotrophin improves the model risk prediction of TCMR. The reference model, consisting of serum creatinine and proteinuria, has an AIC of 190. The risk prediction of the model was significantly improved by the addition of urinary endotrophin (AIC = 183, *P*-value of the *F*-test for the difference between models = .003), but not by the addition of plasma endotrophin (AIC = 192, *P*-value of the *F*-test for the difference between models = .8). The AUC of the ROC curve for the reference model was 0.59 and improved to 0.68 after inclusion of urinary endotrophin (Supplementary Fig. 4).

### Endotrophin staining in kidney biopsies

In order to dissect a possible connection between endotrophin and TCMR, we selected six TCMR biopsies with variable urinary endotrophin excretion along with six biopsies without any sign of rejection. The clinical characteristics of the subset of the KTRs are presented in Supplementary Table 10. We used two different anti-human endotrophin antibodies, the PRO-C6 monoclonal antibody (mAb) recognizing the C-terminal end of ColVIα3 (PRO-C6) and a neo-epitope-specific mAb for the released endotrophin after cleavage by BMP-1 (released endotrophin).

**Figure 2:**
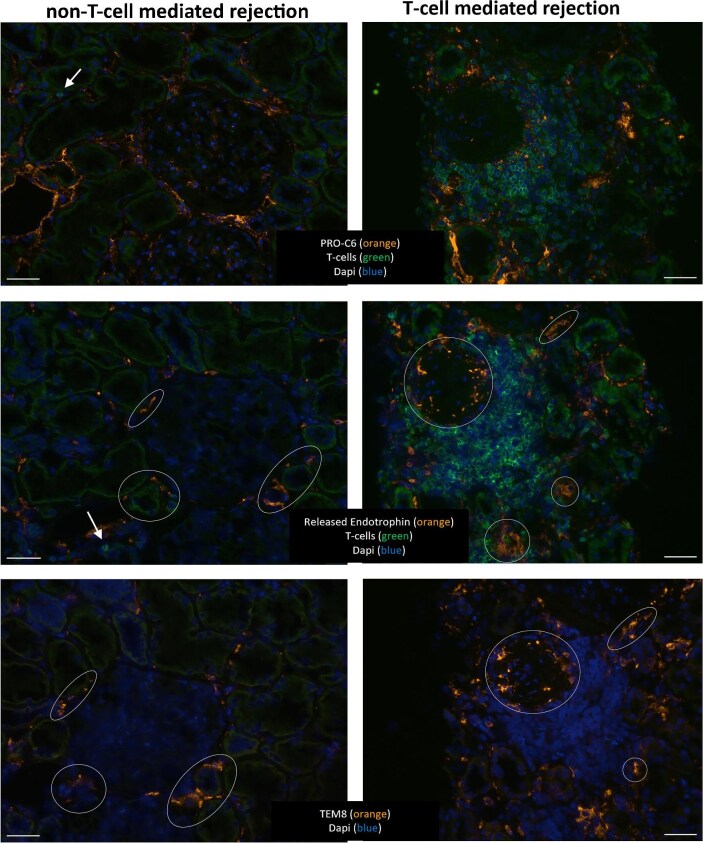
Immunofluorescence staining of T cells (CD3), endotrophin and myofibroblasts (TEM8). The left panel represents a kidney biopsy without TCMR and the right panel is a representative kidney biopsy with TCMR. Sequential sections used three different stainings. Top panel: endotrophin detected by PRO-C6 (orange) with T cells (green). Nuclei (DAPI) are indicated in blue. Middle panel: Released endotrophin (orange) with T cells (green) and nuclei (DAPI, blue). Lower panel: Myofibroblasts (TEM8, orange) and nuclei (DAPI, blue). The top, middle and lower panels show corresponding areas in sequential sections. Bar represents 50 μm. White arrows indicate a single T cell in non-TCMR kidney tissue. Circles in the middle and lower panels indicate corresponding TEM8 and released endotrophin-positive stromal cells, showing myofibroblasts.

Both mAbs were applied in double staining with an antibody recognizing CD3 on T cells. Interestingly, the PRO-C6 mAb showed a variable collagenous fibrous staining pattern within tubulointerstitial areas of the biopsies, whereas glomeruli and vessels were largely negative (Fig. 2, top panel). In contrast, the mAb for the released endotrophin recognized a variable amount of stromal periglomerular and tubulointerstitial cells without any matrix staining (Fig. 2, middle panel). Since TEM-8 has been described as a cellular receptor for endotrophin [25], we stained for TEM-8 in sequential sections and found that both TEM8 and endotrophin were expressed by the same tubulointerstitial stromal cells (Fig. 2, lower panel). According to online Kidney Atlas data [26], kidney cells that express TEM8^+^ are fibroblast-like cells, predominantly myofibroblasts. These data indicate that the released endotrophin-positive interstitial cells are myofibroblasts. Indeed, these TEM8^+^ endotrophin-positive stromal cells proved to be negative for CD31 (endothelial cells) and NG2 (pericytes; data not shown). In the double staining with CD3^+^ T cells, it became clear that the T cells are located within the tubulointerstitium with endotrophin-positive fibres (mAb PRO-C6) and cells (mAb-released endotrophin) mostly around them. Upon the formation of T cell clusters, both the endotrophin fibres (mAb PRO-C6) and cells (mAb-released endotrophin) are around but scarce within the T cell areas (Fig. 2).

**Figure 3: (A):**
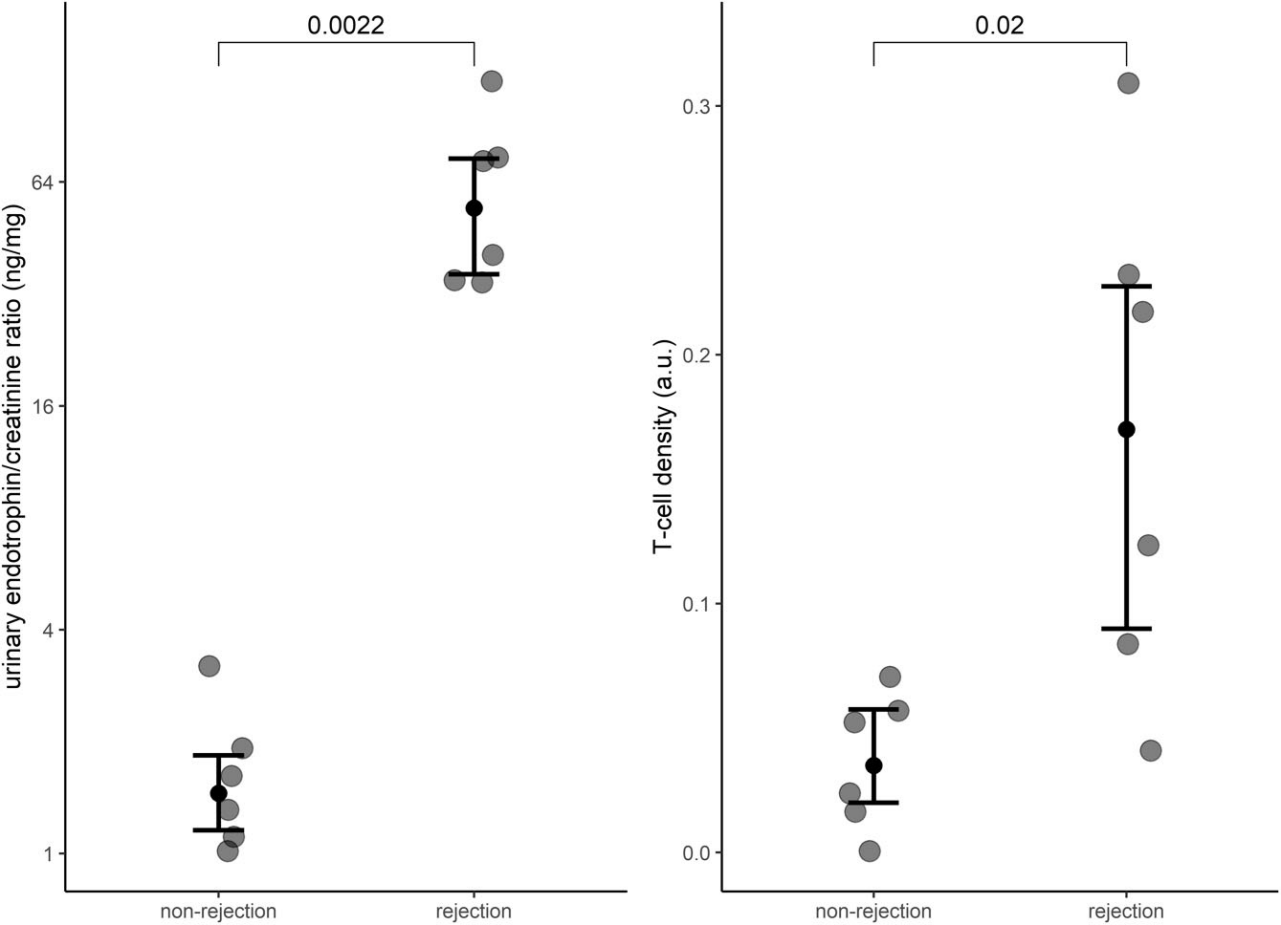
Urinary endotrophin:creatinine ratio and **(B)** T cell density in kidney graft biopsies with (*n* = 6) and without (*n* = 6) TCMR.

The urinary endotrophin level and T cell density were significantly higher in the TCMR compared with the non-TCMR biopsies (Fig. 3). Also, T cell density in the biopsies was correlated with released endotrophin in the biopsies (ρ = 0.61, *P* = .045), urinary endotrophin concentration (ρ = 0.67, *P* = .017) and IFTA score (ρ = 0.61, *P* = .048). Furthermore, both released endotrophin and T cell density in the biopsies correlated with serum creatinine (ρ = 0.69, *P* = .012 and ρ = 0.77, *P* = .004, respectively) and with eGFR (ρ = −0.59, *P* = .045 and ρ = −0.63, *P* = .027, respectively).

## DISCUSSION

In this study, we aimed to evaluate whether endotrophin could be used as a biomarker for fibrogenesis in TCMR among KTRs. We found that urinary endotrophin was associated with higher odds of TCMR, even after adjusting for plasma endotrophin level. In contrast, plasma endotrophin level was not associated with TCMR. This association remained robust across several sensitivity analyses, including analyses that excluded biopsies with BKVAN and other histologic injury phenotypes. Moreover, urinary endotrophin, endotrophin-positive myofibroblasts and IFTA were associated with T cell density in the kidney biopsies. Also, we found that incorporating urinary endotrophin into the currently used clinical parameters for risk assessment, i.e. serum creatinine and proteinuria, significantly improved the predictive value for detecting TCMR. These data suggest that urinary endotrophin may serve as a biomarker for kidney TCMR-mediated fibrogenesis.

In line with previous studies [17, 18], we found that urinary endotrophin significantly correlates with plasma endotrophin, indicating that the endotrophin level in the urine is influenced by its plasma level. However, we found that endotrophin measured in urine but not in plasma was associated with TCMR. It has previously been suggested that urinary endotrophin reflects local pathological changes in the kidney, whereas plasma endotrophin is influenced by systemic factors [16, 19]. As TCMR is a local pathological response within the kidney graft, this may explain why urinary and not plasma endotrophin is associated with TCMR. By adjusting for plasma endotrophin in the association analysis of urinary endotrophin with TCMR, the component of plasma endotrophin appearing in the urine due to filtration from the circulation is mitigated, potentially explaining the strengthening of the association, which now becomes even more reliant on local production of endotrophin in the graft. Our finding is in line with a recent report on liver transplantation showing increased endotrophin related to TCMR as well [27].

One might expect that urinary endotrophin may partly originate from the local production by the native kidney if KTRs still have residual kidney function at the time of transplantation. Nonetheless, we found that urinary endotrophin levels were significantly higher in KTRs without residual kidney function compared with those with residual kidney function. This suggests that native kidneys do not meaningfully contribute to urinary endotrophin levels in KTRs. Interestingly, however, urinary endotrophin was associated with the odds of TCMR only in KTRs with residual kidney function, not in those without. This discrepancy may be explained by differences in donor graft quality between the two groups. In this study, KTRs without residual kidney function more frequently received kidneys from a deceased donor and had longer ischaemia times, a higher prevalence of delayed graft function and worse graft function. Given the poorer graft quality and the likely presence of inflammation and fibrosis prior to transplantation, additional endotrophin release due to TCMR may not substantially increase urinary endotrophin levels beyond existing levels. Therefore, the utility of urinary endotrophin as a non-invasive biomarker for TCMR may be limited to KTRs who received good-quality donor kidneys. Nonetheless, further studies are needed to confirm this hypothesis, as the current study was not primarily designed for this purpose.

Along with the association between urinary endotrophin and TCMR, we also demonstrated the association between T cell density, IFTA and released endotrophin-positive myofibroblasts in the kidney biopsies of KTRs. These findings are in line with spatial transcriptomic data in human kidney transplant rejection showing spatial overlap between T cells and matrix-producing fibroblasts [28]. Also, single-cell analysis in chronic renal transplant rejection demonstrated myofibroblasts with high expression levels of ColVI [29]. In general, it is well recognized that infiltration of leukocytes and a fibrogenic tissue response often go hand in hand [30]. Indeed, TCMR is characterized by a variable amount of IFTA [31], and chemokines such as CCL5 (RANTES) might drive both T cell influx and ColVI synthesis by myofibroblasts [32, 33]. Also, T cells and (myo)fibroblasts, in a reciprocal fashion, might recruit and/or activate each other [34, 35]. Formally, we cannot exclude the possibility that during TCMR, activated T cells secrete factors able to release endotrophin from ColVIα3. In a precision-cut kidney slice model, we evaluated the possibility of endotrophin release by the main T cell protease, namely granzyme B. However, in the supernatants of sections from two different fibrotic kidneys, we could not measure any detectable endotrophin (unpublished data). Although this is not final proof, it makes the possibility of T cell–mediated endotrophin release less likely.

We hypothesize that endotrophin itself could be involved in tissue remodelling related to TCMR. Pro-inflammatory and pro-angiogenic properties of endotrophin have been shown previously [36]. Also, endotrophin has pro-fibrotic properties [37]. Since we found the released endotrophin fragment on TEM8^+^ myofibroblasts, this raised the possibility of fibroblast modulation by endotrophin via the TEM8 receptor, which has previously been proposed as a receptor for endotrophin [25]. Interestingly, TEM8^+^ cancer-associated fibroblasts inhibit anti-tumour T cell cytotoxicity [38]. This raises the question of whether endotrophin might modulate the T cell response in TCMR. In recent years, increasing data have revealed the orchestrating role of stromal cells (mostly fibroblastic cells) on T cell responses, not only in chronic inflammation and anti-tumour responses but also in transplantation [39]. Stromal fibroblastic cells execute such T cell modulation via major histocompatibility complex II–restricted antigen presentation [40], CD40/CD40L interaction [41] and/or via paracrine actions of soluble mediators [42–44] and are mostly tolerogenic. Whether ColVI and/or endotrophin as a proxy for ColVI synthesis down-modulate T cell responses in renal transplantation is not known yet, but is suggested by the finding that in a murine heterotopic cardiac transplantation model mAb ERTR7 (=anti-ColVI) treatment resulted in increased allograft inflammation and rejection [45, 46]. Indeed, ColVI has been shown to trap T cells, thereby inhibiting motility [47]. In vitro findings corroborated the induction of CD8^+^ T cell dysfunction by ColVI [48]. Whether a ColVI/endotrophin response in TCMR represents an adaptive and protective tolerogenic response remains to be seen, but tolerogenic capacity has been shown for different matrix proteins, such as laminin α4 and α5 [49, 50].

The strength of this study is that we measured endotrophin in both plasma and urine and also in a selection of kidney biopsies. By doing this, we could ensure that the association between urinary endotrophin and TCMR was independent of the plasma endotrophin level and that it was related to T cells, myofibroblasts and IFTA in the biopsies. However, the current study also has several limitations to consider. First, the cross-sectional nature of this study does not allow for the assumption of causality. Second, this study was performed in a single centre with an overrepresentation of Caucasian patients, therefore external validation is needed to confirm this finding. Third, we did not have data on other non-invasive novel biomarkers for TCMR. Fourth, we included only indication biopsies. The incidence and phenotype of TCMR differ between protocol and indication biopsies [51]. Fifth, we did not re-evaluate the rejection diagnoses using the latest Banff classification. However, the changes in the Banff classification during the study period are primarily related to ABMR diagnosis criteria rather than TCMR, the main focus of this study. Lastly, residual confounding may still exist despite the number of potentially confounding factors we adjusted for.

## CONCLUSION

This study shows that urinary endotrophin is independently associated with higher odds of having TCMR among KTRs undergoing indication biopsy. Furthermore, this study also shows an intimate relationship among urinary endotrophin, T cells, endotrophin-positive myofibroblasts and IFTA in kidney biopsies. Our data suggest the potential role of urinary endotrophin as a non-invasive biomarker for fibrogenesis in the context of TCMR. Longitudinal studies are needed to confirm this finding. In addition, in vitro experiments adding endotrophin in T cell response assays are needed to better understand the pathomechanistic role of endotrophin in TCMR.

## Supplementary Material

sfaf301_Supplemental_File

## Data Availability

Public sharing of individual participant data was not included in the informed consent forms of the TransplantLines Biobank and Cohort Study, but data will be made available to interested researchers upon reasonable request after approval by the TransplantLines scientific committee (e-mail: datarequest.transplantlines@umcg.nl).

## TRANSPLANTLINES INVESTIGATORS

Coby Annema, Stephan J. L. Bakker, Stefan P. Berger, Hans Blokzijl, Frank A. J. A. Bodewes, Marieke T. de Boer, Kevin Damman, Martin H. de Borst, Arjan Diepstra, Gerard Dijkstra, Caecilia S. E. Doorenbos, Rianne M. Douwes, Michele F. Eisenga, Michiel E. Erasmus, C. Tji Gan, Antonio W. Gomes Neto, Eelko Hak, Bouke G. Hepkema, Jip Jonker, Frank Klont, Tim J. Knobbe, Daan Kremer, Henri G. D. Leuvenink, Willem S. Lexmond, Vincent E. de Meijer, Hubert G. M. Niesters, Gertrude J. Nieuwenhuijs-Moeke, L. Joost van Pelt, Robert A. Pol, Anna M. Posthumus, Adelita V. Ranchor, Jan Stephan F. Sanders, Marion J. Siebelink, Riemer J. H. J. A. Slart, J. Casper Swarte, Daan J. Touw, Marius C. van den Heuvel, Coretta van Leer-Buter, Marco van Londen, Charlotte A. te Velde Keyzer, Erik A. M. Verschuuren, Michel J. Vos and Rinse K. Weersma.
