# Correction to: Urinary endotrophin as a biomarker for T cell–mediated rejection-associated fibrogenesis in kidney transplant recipients

**DOI:** 10.1093/ckj/sfag127

**Published:** 2026-05-08

This is a correction to: Firas F Alkaff, Daan Kremer, Daniel G K Rasmussen, Nadja Sparding, Federica Genovese, Morten Karsdal, Wendy A Dam, Marius C van den Heuvel, Martin Tepel, Olivier Thaunat, TransplantLines Investigators , Stefan P Berger, Jacob van den Born, Stephan J L Bakker, Urinary endotrophin as a biomarker for T cell–mediated rejection-associated fibrogenesis in kidney transplant recipients, *Clinical Kidney Journal*, Volume 18, Issue 11, November 2025, https://doi.org/10.1093/ckj/sfaf301

In the originally published version of the paper an erroneous supplementary data file was linked. That file has been replaced with the corrected supplementary date file.

