# Supplementary material for: Urinary endotrophin as a biomarker for T cell–mediated rejection-associated fibrogenesis in kidney transplant recipients
Source: Clin Kidney J. 2025 Sep 30;18(11):sfaf301. doi: 10.1093/ckj/sfaf301 (PMC12596184; doi:10.1093/ckj/sfaf301)
Supplement: sfaf301_Supplemental_File [file sfaf301_supplemental_file.pdf]

Urinary Endotrophin and T-Cell Mediated Rejection in Kidney Transplant Recipients: Results of the TransplantLines Biobank and Cohort Study

Supplementary files

**Supplementary Figure 1.** Flowchart of the study population selection	Page 2
**Supplementary Figure 2.** Correlation between plasma endotrophin level and urinary endotrophin/creatinine ratio in kidney transplant recipients undergoing indication biopsy.	Page 3
**Supplementary Figure 3.** Plasma and urinary endotrophin levels based on the types of rejection.	Page 4
**Supplementary Figure 4.** ROC curve of the reference model before and after the addition of plasma or urinary endotrophin for the prediction of T-cell mediated rejection.	Page 5
**Supplementary Table 1.** STROBE Statement—Checklist of items that should be included in reports of ***cross-sectional studies***	Page 6
**Supplementary Table 2.** Detailed double immunofluorescence staining procedure on human kidney biopsy cryosections	Page 9
**Supplementary Table 3.** Detailed TEM8 immunofluorescence staining procedure on human kidney biopsy cryosections	Page 11
**Supplementary Table 4.** Logistic regression analyses of the association of endotrophin with the odds of TCMR in KTR that underwent biopsies ≥ 1 month after transplantation	Page 12
**Supplementary Table 5.** Logistic regression analyses of the association of endotrophin with the odds of TCMR in KTR that underwent biopsies ≥ 3 months after transplantation	Page 13
**Supplementary Table 6.** Logistic regression analyses of the association of endotrophin with the odds of TCMR in KTR without BK virus-associated nephropathy	Page 14
**Supplementary Table 7.** Logistic regression analyses of the association of endotrophin with the odds of TCMR in KTR without BK virus-associated nephropathy, antibody-mediated rejection, and transplant glomerulopathy	Page 15
**Supplementary Table 8.** Baseline characteristics of the kidney transplant recipients stratified based on the presence of residual kidney function at the time of transplantation	Page 16
**Supplementary Table 9.** Logistic regression analyses of the association of endotrophin with the odds of TCMR among KTR with and without residual kidney function at the time of transplantation	Page 18
**Supplementary Table 10.** Individual clinical data on the subset of kidney transplant recipients in which the biopsy was used for immunofluorescent staining	Page 19

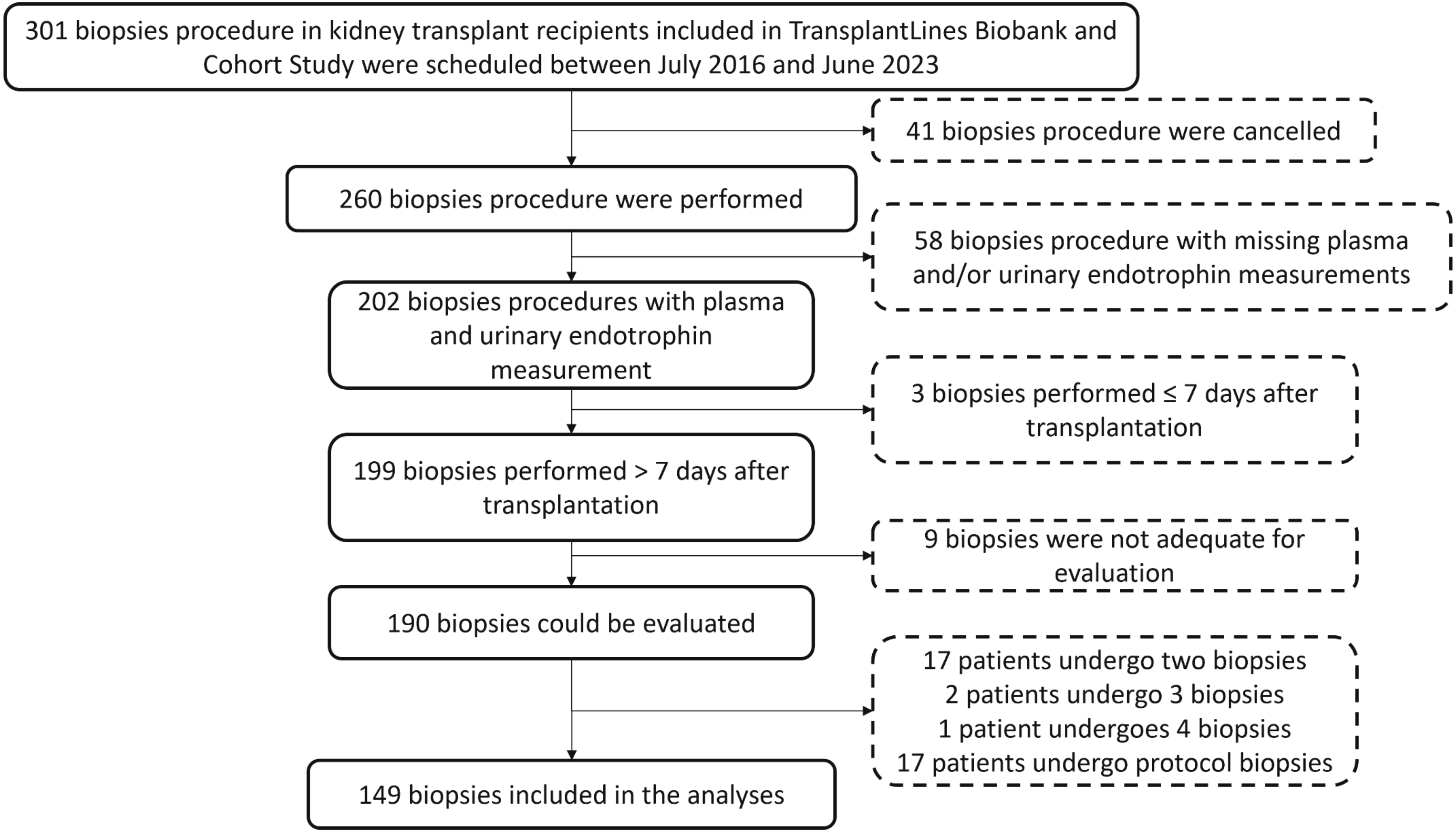

Supplementary Figure 1. Flowchart of the study population selection

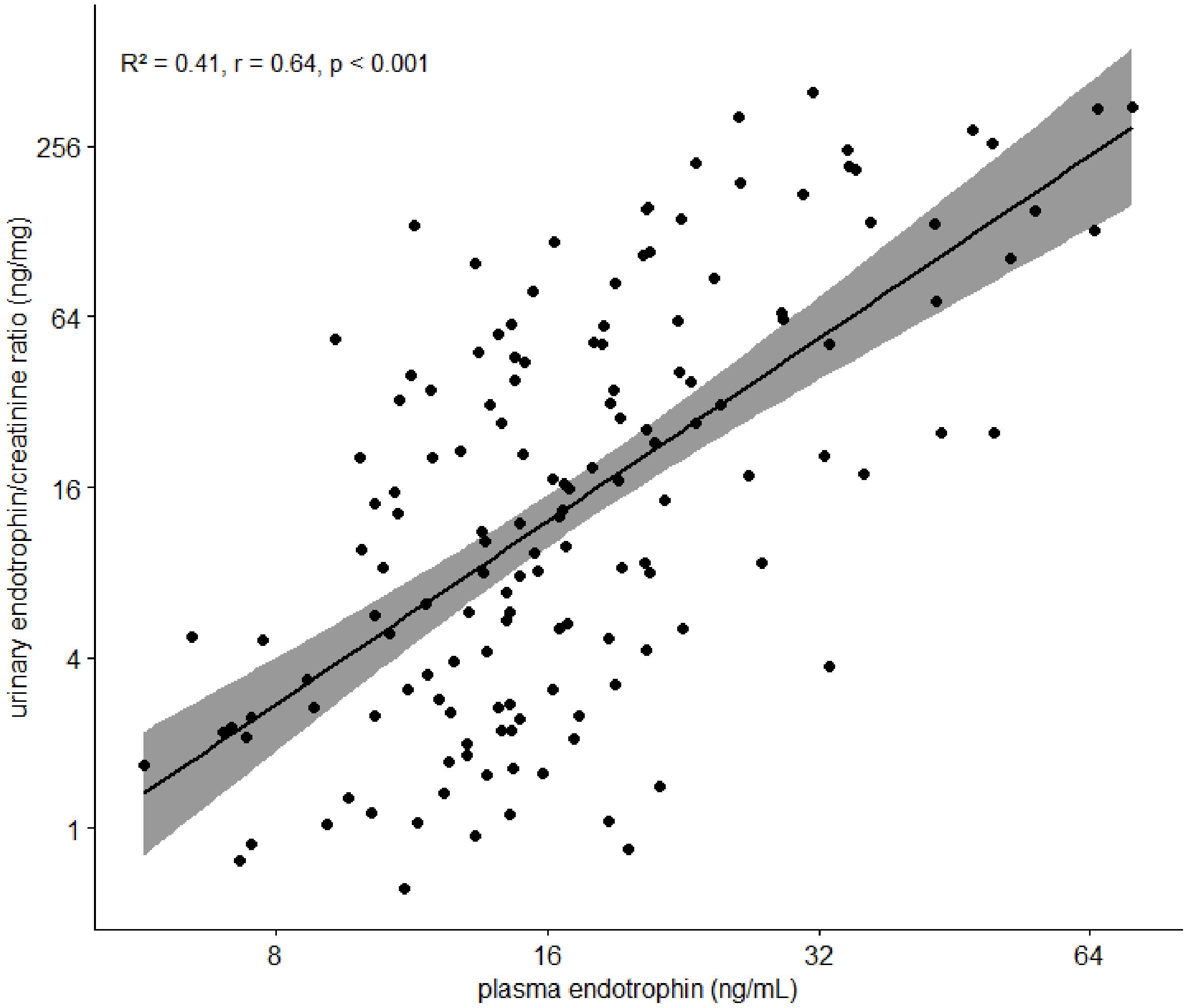

Supplementary Figure 2. Correlation between plasma endotrophin level and urinary endotrophin/creatinine ratio in kidney transplant recipients undergoing indication biopsy.

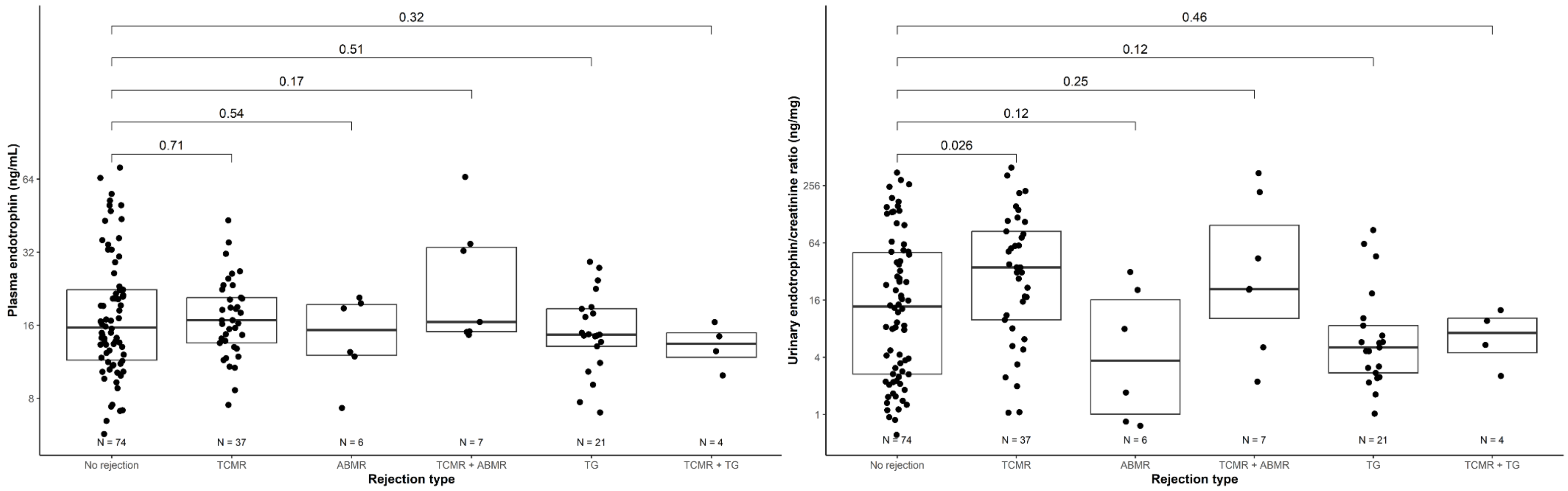

Supplementary Figure 3. Plasma and urinary endotrophin levels based on the types of rejection.
The boxplot indicates the median [interquartile range]. ABMR, antibody-mediated rejection; TCMR, T-cell-mediated rejection; TG, transplant glomerulopathy

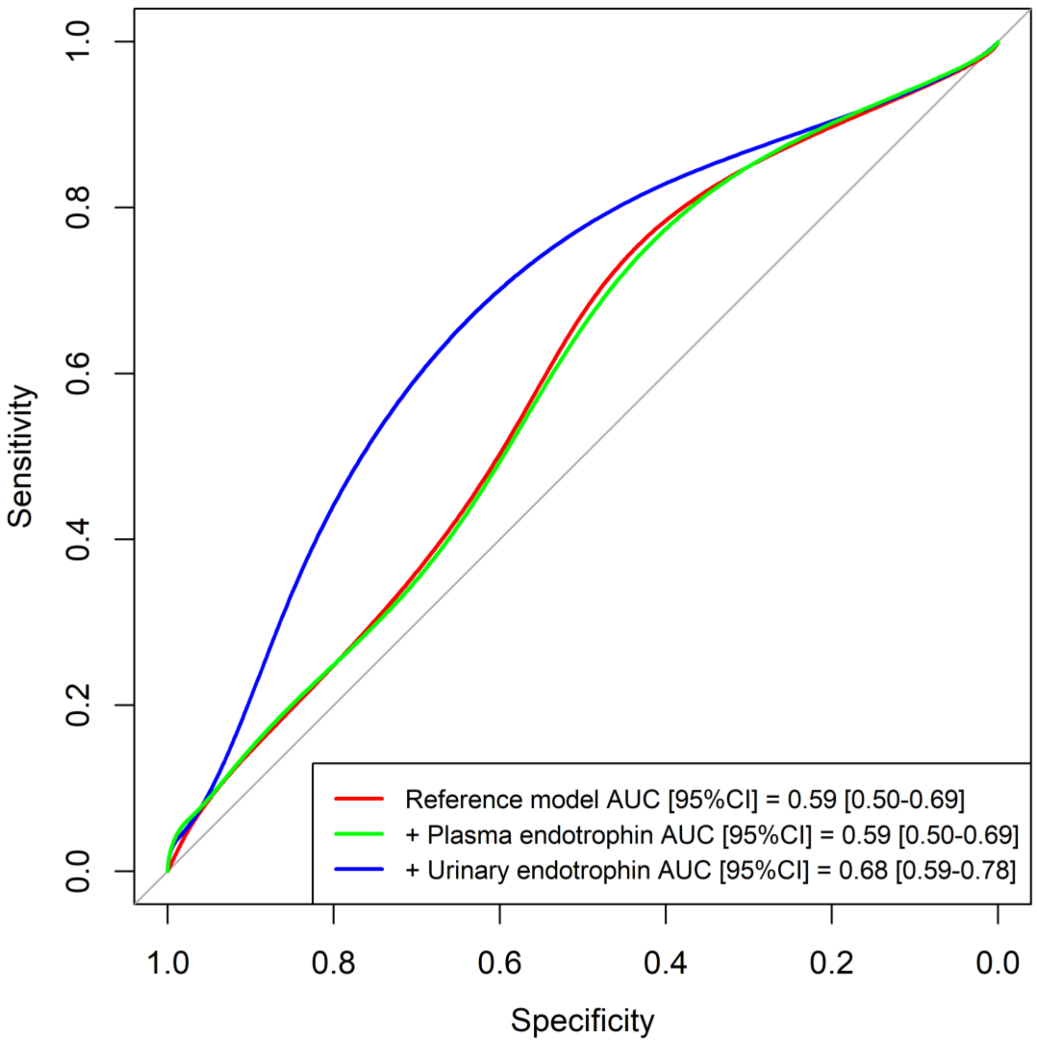

Supplementary Figure 4. ROC curve of the reference model before and after the addition of plasma or urinary endotrophin for the prediction of T-cell mediated rejection. The reference model was composed of serum creatinine and proteinuria. AUC, area under the curve; ROC, receiver operator characteristic; 95%CI, 95% confidence interval.

Supplementary table 1. STROBE Statement—Checklist of items that should be included in reports of ***cross-sectional studies***

	Item No	Recommendation	Page No
**Title and abstract**	1	(*a*) Indicate the study's design with a commonly used term in the title or the abstract	3
		(*b*) Provide in the abstract an informative and balanced summary of what was done and what was found	3
**Introduction**			
Background/rationale	2	Explain the scientific background and rationale for the investigation being reported	5-6
Objectives	3	State specific objectives, including any prespecified hypotheses	6
**Methods**			
Study design	4	Present key elements of study design early in the paper	7
Setting	5	Describe the setting, locations, and relevant dates, including periods of recruitment, exposure, follow-up, and data collection	7
Participants	6	(*a*) Give the eligibility criteria, and the sources and methods of selection of participants	7
Variables	7	Clearly define all outcomes, exposures, predictors, potential confounders, and effect modifiers. Give diagnostic criteria, if applicable	8-10
Data sources/ measurement	8*	For each variable of interest, give sources of data and details of methods of assessment (measurement). Describe comparability of assessment methods if there is more than one group	7-8
Bias	9	Describe any efforts to address potential sources of bias	7
Study size	10	Explain how the study size was arrived at	7
Quantitative variables	11	Explain how quantitative variables were handled in the analyses. If applicable, describe which groupings were chosen and why	9

Statistical methods	12	(*a*) Describe all statistical methods, including those used to control for confounding	9-11
		(*b*) Describe any methods used to examine subgroups and interactions	10-11
		(*c*) Explain how missing data were addressed	10
		(*d*) If applicable, describe analytical methods taking account of sampling strategy	N/A
		(*e*) Describe any sensitivity analyses	10
**Results**			
Participants	13*	(a) Report numbers of individuals at each stage of study—eg numbers potentially eligible, examined for eligibility, confirmed eligible, included in the study, completing follow-up, and analysed	Supplementary Figure 1
		(b) Give reasons for non-participation at each stage	Supplementary Figure 1
		(c) Consider use of a flow diagram	Supplementary Figure 1
Descriptive data	14*	(a) Give characteristics of study participants (eg demographic, clinical, social) and information on exposures and potential confounders	12
		(b) Indicate number of participants with missing data for each variable of interest	Table 1
Outcome data	15*	Report numbers of outcome events or summary measures	12
Main results	16	(*a*) Give unadjusted estimates and, if applicable, confounder-adjusted estimates and their precision (eg, 95% confidence interval). Make clear which confounders were adjusted for and why they were included	12-13, Table 2
		(*b*) Report category boundaries when continuous variables were categorized	N/A

		(*c*) If relevant, consider translating estimates of relative risk into absolute risk for a meaningful time period	N/A
Other analyses	17	Report other analyses done—eg analyses of subgroups and interactions, and sensitivity analyses	13-14
**Discussion**			
Key results	18	Summarise key results with reference to study objectives	16
Limitations	19	Discuss limitations of the study, taking into account sources of potential bias or imprecision. Discuss both direction and magnitude of any potential bias	19
Interpretation	20	Give a cautious overall interpretation of results considering objectives, limitations, multiplicity of analyses, results from similar studies, and other relevant evidence	16-19
Generalisability	21	Discuss the generalisability (external validity) of the study results	19
**Other information**			
Funding	22	Give the source of funding and the role of the funders for the present study and, if applicable, for the original study on which the present article is based	20-21

Supplementary Table 2. Detailed double immunofluorescence staining procedure on human kidney biopsy cryosections

Procedure	PRO-C6/CD3	Released endotrophin/CD3
**Fixation**	4% formaldehyde for 10 minutes	Acetone (-20ºC) for 10 minutes
**Peroxidase inactivation**	H_2O_2 (1:1000 dilution, Sigma-Aldrich #216763) for 30 minutes	
**1st background Blocking**	1%BSA in PBS for 30 minutes	
**Endogenous biotin blocking**	Avidin block for 10 minutes, followed by Biotin block for 10 minutes (Vector #SP-2001)	Streptavidin block for 15 minutes, followed by Biotin block for 15 minutes (Vector #SP-2002)
	**First staining**	
**Primary antibody**	Mouse mAb anti-human PRO-C6 (final concentration 0.0625 µg/ml, Nordic Bioscience) overnight at 4ºC	Mouse mAb anti-human released endotrophin (final concentration 0.142 µg/ml, Nordic Bioscience) overnight at 4ºC
**2nd background blocking**	10% normal human serum for 30 minutes	
**Secondary antibody**	Goat anti-mouse IgG HRP (1:100 dilution, DAKO #P0447) for 30 minutes + 5% normal human serum + 1% BSA	
**Tertiary antibody**	Donkey anti-goat IgG HRP (1:100 dilution, Southern Biotech #6420-05) for 30 minutes + 5% normal human serum + 1%BSA	
**HRP Signal amplification**	TSA Tyramide-TRITC (1:50 dilution, Akoya Biosciences # SAT702001EA) in amplification buffer for 10 minutes	
	**Second (CD3) staining**	
**Primary antibody**	Rabbit pAb anti-human CD3 (1:50 dilution, DAKO #A0452) for 60 minutes	

**3rd background blocking**	10% normal goat serum in 1%BSA for 30 minutes
**Secondary antibody**	Goat anti-rabbit Biotin (1:200 dilution, DAKO #E0432) for 30 minutes + 5% normal human serum + 1%BSA
**Tertiary antibody**	Streptavidine FITC (1:300, eBioscience #11-4317-87) for 30 minutes + 5% normal human serum + 1%BSA
**Nuclear staining**	DAPI (Sigma-Aldrich #D9542) for 10 minutes
**Embedment**	Citifluor (Haffield PA, USA)

Unless stated otherwise, the incubations were done at room temperature. Sections were washed with PBS in between the incubation steps.

Supplementary Table 3. Detailed TEM8 immunofluorescence staining procedure on human kidney biopsy cryosections

**Fixation**	Acetone (-20ºC) for 10 minutes
**Peroxidase inactivation**	H_2O_2 (1:1000 dilution, Sigma-Aldrich #216763) for 30 minutes
**1st background Blocking**	1%BSA in PBS for 30 minutes
**Primary antibody**	Rabbit pAb anti-human TEM8 (1:2000 dilution, Bioss #bs-15583R) overnight at 4ºC
**2nd background blocking**	10% normal human serum in PBS for 30 minutes
**Secondary antibody**	Goat anti-rabbit IgG HRP (1:100 dilution, DAKO #P0448) for 30 minutes + 5% normal human serum + 1%BSA
**Tertiary antibody**	Rabbit anti-goat IgG HRP (1:100 dilution, DAKO #P0449) for 30 minutes + 5% normal human serum + 1%BSA
**HRP Signal amplification**	TSA Tyramide-TRITC (1:50 dilution, Akoya Biosciences # SAT702001EA) in amplification buffer for 10 minutes
**Nuclear staining**	DAPI (Sigma-Aldrich #D9542) for 10 minutes
**Embedment**	Citifluor (Haffield PA, USA)

Unless stated otherwise, the incubations were done at room temperature. Sections were washed with PBS in between the incubation steps.

Supplementary Table 4. Logistic regression analyses of the association of endotrophin with the odds of TCMR in KTR that underwent biopsies ≥ 1 month after transplantation

Model	Plasma endotrophin		Urinary endotrophin/creatinine ratio	
	OR per doubling [95% CI]	P-value	OR per doubling [95% CI]	P-value
Crude	1.60 [0.90-2.84]	0.1	1.37 [1.16-1.62]	<0.001
Model 1	1.58 [0.88-2.82]	0.1	1.37 [1.15-1.62]	<0.001
Model 2	1.72 [0.95-3.14]	0.075	1.36 [1.14-1.61]	<0.001
Model 3	1.09 [0.50-2.38]	0.8	1.32 [1.07-1.63]	0.009
Model 4	1.11 [0.51-2.45]	0.8	1.35 [1.09-1.66]	0.006
Model 5	1.07 [0.48-2.36]	0.9	1.33 [1.07-1.65]	0.009
Model 6	-	-	1.38 [1.10-1.74]	0.006

Of 149 KTR in the primary analysis, 13 were excluded, leaving 136 KTR for this sensitivity analysis. Of them, 46 (34%) KTR had TCMR. Logistic regression analyses were performed to assess the association of plasma endotrophin level and urinary endotrophin/creatinine ratio with the odds of TCMR. Model 1 was adjusted for age and sex. Model 2 was further adjusted for time after transplantation. Model 3 was further adjusted for the estimated glomerular filtration rate based on the creatinine-based CKD-EPI formula. Model 4 was further adjusted for $\log_2$ urinary protein/creatinine ratio. Model 5 was further adjusted for C-reactive protein level. Model 6 was further adjusted for $\log_2$ plasma endotrophin level.
95%CI, 95% confidence interval; KTR, kidney transplant recipients; OR, odds ratio; TCMR, T-cell mediated rejection including borderline rejection.

Supplementary Table 5. Logistic regression analyses of the association of endotrophin with the odds of TCMR in KTR that underwent biopsies ≥ 3 months after transplantation

Model	Plasma endotrophin		Urinary endotrophin/creatinine ratio	
	OR per doubling [95% CI]	P-value	OR per doubling [95% CI]	P-value
Crude	1.46 [0.81-2.61]	0.2	1.35 [1.14-1.61]	<0.001
Model 1	1.46 [0.81-2.63]	0.2	1.35 [1.14-1.61]	<0.001
Model 2	1.57 [0.85-2.88]	0.2	1.34 [1.13-1.60]	<0.001
Model 3	0.85 [0.38-1.92]	0.7	1.28 [1.03-1.59]	0.024
Model 4	0.87 [0.38-1.99]	0.7	1.31 [1.05-1.63]	0.017
Model 5	0.87 [0.38-1.97]	0.7	1.30 [1.04-1.63]	0.021
Model 6	-	-	1.39 [1.09-1.77]	0.009

Of 149 KTR in the primary analysis, 25 were excluded, leaving 124 KTR for this sensitivity analysis. Of them, 44 (35%) KTR had TCMR. Logistic regression analyses were performed to assess the association of plasma endotrophin level and urinary endotrophin/creatinine ratio with the odds of TCMR. Model 1 was adjusted for age and sex. Model 2 was further adjusted for time after transplantation. Model 3 was further adjusted for the estimated glomerular filtration rate based on the creatinine-based CKD-EPI formula. Model 4 was further adjusted for $\log_2$ urinary protein/creatinine ratio. Model 5 was further adjusted for C-reactive protein level. Model 6 was further adjusted for $\log_2$ plasma endotrophin level.
95%CI, 95% confidence interval; KTR, kidney transplant recipients; OR, odds ratio; TCMR, T-cell mediated rejection including borderline rejection.

Supplementary Table 6. Logistic regression analyses of the association of endotrophin with the odds of TCMR in KTR without BK virus-associated nephropathy

Model	Plasma endotrophin		Urinary endotrophin/creatinine ratio	
	OR per doubling [95% CI]	P-value	OR per doubling [95% CI]	P-value
Crude	1.10 [0.69-1.76]	0.7	1.25 [1.07-1.45]	<0.001
Model 1	1.08 [0.67-1.75]	0.7	1.25 [1.07-1.45]	<0.001
Model 2	1.04 [0.64-1.69]	0.9	1.22 [1.05-1.42]	0.009
Model 3	0.67 [0.33-1.36]	0.3	1.26 [1.03-1.52]	0.022
Model 4	0.72 [0.35-1.47]	0.4	1.30 [1.06-1.59]	0.011
Model 5	0.90 [0.42-1.94]	0.8	1.33 [1.08-1.63]	0.007
Model 6	-	-	1.41 [1.13-1.77]	0.003

Of 149 KTR in the primary analysis, 5 were excluded due to the presence of BK virus-associated nephropathy, leaving 144 KTR for this sensitivity analysis. Of them, 48 (33%) KTR had TCMR. Logistic regression analyses were performed to assess the association of plasma endotrophin level and urinary endotrophin/creatinine ratio with the odds of TCMR. Model 1 was adjusted for age and sex. Model 2 was further adjusted for time after transplantation. Model 3 was further adjusted for the estimated glomerular filtration rate based on the creatinine-based CKD-EPI formula. Model 4 was further adjusted for $\log_2$ urinary protein/creatinine ratio. Model 5 was further adjusted for C-reactive protein level. Model 6 was further adjusted for $\log_2$ plasma endotrophin level.
95%CI, 95% confidence interval; KTR, kidney transplant recipients; OR, odds ratio; TCMR, T-cell mediated rejection including borderline rejection.

Supplementary Table 7. Logistic regression analyses of the association of endotrophin with the odds of TCMR in KTR without BK virus-associated nephropathy, antibody-mediated rejection, and transplant glomerulopathy.

Model	Plasma endotrophin		Urinary endotrophin/creatinine ratio	
	OR per doubling [95% CI]	P-value	OR per doubling [95% CI]	P-value
Crude	0.90 [0-53-1.53]	0.7	1.20 [1.02-1.42]	0.032
Model 1	0.86 [0.49-1.49]	0.6	1.20 [1.01-1.42]	0.036
Model 2	0.83 [0.48-1.46]	0.5	1.19 [1.00-1.42]	0.047
Model 3	0.49 [0.22-1.12]	0.092	1.23 [0.99-1.53]	0.056
Model 4	0.51 [0.22-1.16]	0.1	1.30 [1.03-1.64]	0.026
Model 5	0.50 [0.21-1.19]	0.1	1.31 [1.04-1.65]	0.024
Model 6	-	-	1.53 [1.16-2.01]	0.003

Of 149 KTR in the primary analysis, 43 were excluded due to the presence of BK virus-associated nephropathy (n = 5), antibody-mediated rejection (n = 13), and transplant glomerulopathy (n = 25), leaving 106 KTR for this sensitivity analysis. Of them, 37 (35%) KTR had TCMR. Logistic regression analyses were performed to assess the association of plasma endotrophin level and urinary endotrophin/creatinine ratio with the odds of TCMR. Model 1 was adjusted for age and sex. Model 2 was further adjusted for time after transplantation. Model 3 was further adjusted for the estimated glomerular filtration rate based on the creatinine-based CKD-EPI formula. Model 4 was further adjusted for $\log_2$ urinary protein/creatinine ratio. Model 5 was further adjusted for C-reactive protein level. Model 6 was further adjusted for $\log_2$ plasma endotrophin level.
95%CI, 95% confidence interval; KTR, kidney transplant recipients; OR, odds ratio; TCMR, T-cell mediated rejection including borderline rejection.

Supplementary Table 8. Baseline characteristics of the kidney transplant recipients stratified based on the presence of residual kidney function at the time of transplantation

	**Residual kidney function**<br><br>**N = 106**	**Without residual kidney function**<br><br>**N = 42**	**P-value**
Plasma endotrophin level, ng/mL	15 [12-21]	19 [14-26]	0.060
Urinary Endotrophin/creatinine ratio, ng/mg	**10 [2-38]**	**28 [5-87]**	**0.019**
TCMR rejection, n (%)	37 (35)	11 (26)	0.3
**Demographics**			
Female sex, n (%)	40 (38)	17 (40)	0.9
Age, years	53 ± 16	54 ± 13	0.6
Time from transplantation, months	21 [4-77]	27 [6-85]	0.5
**Transplant-related characteristics**			
Preemptive transplantation, n (%)	**48 (45)**	**0 (0)**	**<0.001**
First kidney transplantation, n (%)	80 (75)	26 (62)	0.11
Total HLA (A-B-DR) mismatch	3 [2-4]	3 [1-4]	0.065
Cold ischemic time, minutes	**185 [152-623]**	**569 [180-910]**	**0.001**

Living donor, n (%)	**69 (65)**	**16 (38)**	**0.003**
Donor age, years	51 ± 14	51 ± 14	1.0
Delayed graft function, n (%)	**15 (14)**	**14 (33)**	**0.012**
**Immunosuppressive medication**			
Use of calcineurin inhibitors, n (%)	93 (93)	38 (97)	0.4
Use of proliferation inhibitors, n (%)	77 (77)	34 (87)	0.2
Use of mTOR inhibitors, n (%)	9 (9.0)	1 (2.6)	0.3
**Laboratory evaluation**			
CRP, mg/L	**3 [1-5]**	**4 [2-13]**	**0.010**
Serum creatinine, µmol/L	**179 [152-264]**	**214 [172-304]**	**0.014**
eGFR, mL/min/1.73m^2	**32 ± 16**	**26 ± 13**	**0.022**
Urinary creatinine, mg/dL	71 [48-103]	62 [41-104]	0.4
Urinary protein/creatinine ratio, g/10mmol	0.01 [0.00-0.02]	0.01 [0.00-0.03]	0.4

Immunosuppressive medication was missing in 10 (6.7%) subjects, IFTA was missing in 10 (6.7%) subjects, CRP was missing in 29 (19.5%) subjects, and protein/creatinine ratio was missing in 13 (8.7%) subjects. CRP, C-reactive protein; eGFR, estimated glomerular filtration rate according to creatinine-based CKD-EPI formula; IFTA, interstitial fibrosis/tubular atrophy; mTOR, mechanistic target of rapamycin; TCMR, T-cell mediated rejection including borderline rejection. P-value < 0.05 was considered statistically significant.

Supplementary Table 9. Logistic regression analyses of the association of endotrophin with the odds of TCMR among KTR with and without residual kidney function at the time of transplantation

	With residual kidney function (N = 106)				Without residual kidney function (N = 42)			
Model	Plasma endotrophin		Urinary endotrophin/creatinine ratio		Plasma endotrophin		Urinary endotrophin/creatinine ratio	
	OR per doubling [95% CI]	P-value	OR per doubling [95% CI]	P-value	OR per doubling [95% CI]	P-value	OR per doubling [95% CI]	P-value
Crude	1.31 [0.75-2.28]	0.3	1.33 [1.11-1.59]	<0.001	0.81 [0.29-2.21]	0.7	1.13 [0.83-1.54]	0.5
Model 1	1.30 [0.74-2.27]	0.4	1.33 [1.11-1.59]	<0.001	0.66 [0.22-2.04]	0.5	1.12 [0.81-1.54]	0.5
Model 2	1.27 [0.73-2.23]	0.4	1.32 [1.10-1.59]	0.003	0.61 [0.17-2.20]	0.5	1.11 [0.79-1.56]	0.5
Model 3	0.77 [0.33-1.80]	0.6	1.34 [1.06-1.69]	0.013	0.39 [0.08-1.78]	0.2	1.07 [0.71-1.60]	0.7
Model 4	0.91 [0.38-2.17]	0.8	1.49 [1.15-1.93]	0.003	0.30 [0.06-1.58]	0.2	1.06 [0.71-1.60]	0.8
Model 5	1.04 [0.42-2.61]	0.9	1.50 [1.15-1.94]	0.002	0.30 [0.06-1.49]	0.1	1.08 [0.71-1.64]	0.7
Model 6	-	-	1.55 [1.18-2.04]	0.002	-	-	1.30 [0.77-2.20]	0.3

There were 37 (35%) KTR with residual urine production and 11 (26%) KTR without residual urine production that had TCMR. Logistic regression analyses were performed to assess the association of plasma endotrophin level and urinary endotrophin/creatinine ratio with the odds of having TCMR. Model 1 was adjusted for age and sex. Model 2 was further adjusted for time after transplantation. Model 3 was further adjusted for the estimated glomerular filtration rate based on the creatinine-based CKD-EPI formula. Model 4 was further adjusted for log2 urinary protein/creatinine ratio. Model 5 was further adjusted for C-reactive protein level. Model 6 was further adjusted for log2 plasma endotrophin level. 95%CI, 95% confidence interval; KTR, kidney transplant recipients; OR, odds ratio; TCMR, T-cell mediated rejection including borderline rejection.

Supplementary Table 10. Individual clinical data on the subset of kidney transplant recipients in which the biopsy was used for immunofluorescent staining

Number	Biopsy type	TCMR	Months after transplantation	Serum creatinine (µmol/L)	Urinary endotrophin/creatinine ratio (ng/mg)
#1	Indication	Yes	3	159	396
#2	Indication	Yes	12	605	213.3
#3	Indication	Yes	15	250	218.5
#4	Indication	Yes	52	363	83.6
#5	Indication	Yes	52	326	84.5
#6	Indication	Yes	131	384	102.6
#7	Indication	No	3	272	4.3
#8	Indication	No	6	120	1.1
#9	Indication	No	13	235	1.4
#10	Indication	No	13	210	1.8
#11	Indication	No	50	166	1.0
#12	Indication	No	138	143	2.3
